# Correlation between Results of Semi-Quantitative and Quantitative Tests for Hepatitis B Virus Surface Antigen among Patients Achieving Viral Suppression with Antiviral Treatment

**DOI:** 10.3390/diagnostics12071757

**Published:** 2022-07-20

**Authors:** Goh Eun Chung, Ju Yeon Kim, Hyunjae Shin, Ji Hoon Hong, Moon Haeng Hur, Heejin Cho, Min Kyung Park, Na Ryung Choi, Jihye Kim, Yun Bin Lee, Eun Ju Cho, Su Jong Yu, Yoon Jun Kim, Jung-Hwan Yoon, Jeong-Hoon Lee

**Affiliations:** 1 Department of Internal Medicine, Healthcare Research Institute, Gangnam Healthcare Center, Seoul National University Hospital, Seoul 03080, Korea; gohwom@snu.ac.kr; 2 Department of Internal Medicine, Liver Research Institute, Seoul National University College of Medicine, Seoul 03080, Korea; kimjuyean@naver.com (J.Y.K.); yhstella@gmail.com (H.S.); jhhongmd@gmail.com (J.H.H.); mhhur@snu.ac.kr (M.H.H.); genezzang@gmail.com (H.C.); alsrud627@snu.ac.kr (M.K.P.); lemonsmilk87@hanmail.net (N.R.C.); no1spina@naver.com (J.K.); yblee@snu.ac.kr (Y.B.L.); creatioex@gmail.com (E.J.C.); sujongyu@gmail.com (S.J.Y.); yoonjun@snu.ac.kr (Y.J.K.); yoonjh@snu.ac.kr (J.-H.Y.)

**Keywords:** hepatitis B virus (HBV), hepatitis B surface antigen (HBsAg), hook effect

## Abstract

*Background*: Hepatitis B virus (HBV) infection remains a threat to global public health. Serum hepatitis B surface antigen (HBsAg) has been used in screening for HBV infection. Quantitative HBsAg assays are useful for monitoring the natural history of HBV infection and its response to therapy. The aim of this study was to determine the relationship between quantitative (qHBsAg; IU/mL) and semi-quantitative (sqHBsAg; signal-to-cutoff ratio [S/Co]) HBsAg titers in patients with chronic hepatitis B (CHB). *Methods*: We retrospectively included 284 samples with HBV DNA < 20 IU/mL from patients who had simultaneous qHBsAg (using electrochemiluminescence assay) and sqHBsAg tests. Patients were grouped according to their serum HBV-envelope antigen (HBeAg) status (HBeAg-negative, *n* = 239 and HBeAg-positive, *n* = 45). The Spearman test was used to analyze the correlation between the quantitative and semi-quantitative assays. *Results*: There was a significant linear correlation between sqHBsAg and qHBsAg in the HBeAg-negative patients (qHBsAg [IU/mL] = 0.0094 × sqHBsAg [S/Co]^1.323^; adjusted R^2^ = 0.8445; *p* < 0.001). There was a substantial hook effect in the assays from the HBeAg-positive patients, so we performed a stratified analysis according to qHBsAg <1000 IU/mL or ≥1000 IU/mL and found a significant positive linear correlation between sqHBsAg S/Co and qHBsAg (qHBsAg [IU/mL] = 0.072 × sqHBsAg [S/Co]^1.331^; adjusted R^2^ = 0.7878; *p* < 0.001) in HBeAg-positive patients with qHBsAg titers of <1000 IU/mL and a significant negative correlation in HBeAg-positive patients with qHBsAg titers of ≥1000 IU/mL (qHBsAg [IU/mL] = 8.987 × 10^14^ × sqHBsAg [S/Co]^−3.175^; adjusted R^2^ = 0.6350; *p* < 0.001). *Conclusions*: There was a highly linear, positive correlation between qHBsAg and sqHBsAg in HBeAg-negative CHB patients. The hook effect led to a negative correlation in HBeAg-positive CHB patients with qHBsAg titers ≥1000 IU/mL.

## 1. Introduction

Chronic hepatitis B (CHB) is a global health concern known for potentially serious outcomes including cirrhosis, hepatic decompensation, and hepatocellular carcinoma. The primary goals of therapy in CHB are the prevention of disease progression and prolongation of survival through the long-term suppression of the virus, as indicated by the monitoring of serum hepatitis B surface antigen (HBsAg), hepatitis B virus (HBV)-DNA, and covalently closed circular DNA (cccDNA) [1,2,3]. Serum quantitative HBsAg (qHBsAg; IU/mL) titer is accepted as a surrogate marker for cccDNA in infected hepatocytes and as a marker of the host immune control of HBV infection [4]. In patients with low viral loads, higher qHBsAg levels have been shown to predict a greater risk of hepatocellular carcinoma development and disease progression [5,6]. 

The presence of HBsAg in the serum is the classic hallmark of HBV infection before serum HBV DNA is detectable. In clinical practice, HBsAg testing has long served as a diagnostic marker for individuals infected with HBV. Currently, qHBsAg is methodologically feasible and is reported to be useful for monitoring the natural history of the disease and its response to therapy [7,8]. The correlation between serum qHBsAg and HBV DNA concentrations and cccDNA has also been investigated [9]. In addition, qHBsAg monitoring by immunoradiometric assay methods might be useful for differentiating the phases of CHB and stages of chronic liver disease [10]. The qHBsAg assay has been promoted as a reliable, reproducible, sensitive, and specific method for HBsAg quantitation as well as detection [11]. However, it was introduced more recently (in 2011), and the price is relatively high [12]. On the other hand, semi-quantitative HBsAg (sqHBsAg; signal-to-cutoff ratio [S/Co]) measurement has a relatively low price and has been tested since the 1970s, and far more data regarding this assay method have been accumulated in clinical practice [13]. As such, if the conversion from sqHBsAg to qHBsAg is reliable and feasible, it may be helpful for evaluating the effect of qHBsAg on long-term outcomes. Although the qHBsAg assay is currently widely used, this transformation needs to be developed in a way that incorporates the results of previous sqHBsAg testing. Therefore, this study aimed to examine the correlation between qHBsAg and sqHBsAg titers in patients with CHB. 

## 2. Patients and Methods

### 2.1. Study Population

This retrospective cohort study included 293 CHB patients who had simultaneous qHBsAg and sqHBsAg titers performed at Seoul National University Hospital (Seoul, Republic of Korea) from 1 January 2013 to 31 January 2021. Patients with HBV DNA > 20 IU/mL were excluded (*n* = 9) and grouped according to HBV envelope antigen (HBeAg) status. There were 239 in the HBeAg-negative group and 45 in the HBeAg-positive group. 

This study was conducted following the ethical guidelines of the Declaration of Helsinki and was approved by the Institutional Review Board of Seoul National University Hospital (IRB No H-1903-034-1016). Informed consent was waived by the Institutional Review Board because of the retrospective and anonymized nature of the data.

### 2.2. Laboratory Assays

The S/Co values for the sqHBsAg assay were determined by the ratio of a signal value obtained from a control group and the signal value from the patient sample, where S_cutoff_ = (A × S_neg_) + (B × S_pos_) + C, with the values of the constants provided by the manufacturers. In this study, the sqHBsAg values were obtained by using the Architect HBsAg assay (Abbott Diagnostics, Lake Forest, IL, USA) according to the manufacturer’s recommendations. In brief, after 1:100 dilution with the serum diluent, samples with HBsAg levels >250 IU/mL were retested at a dilution of 1:500 and 1:1000 until the final concentration was obtained [14]. The value was presented in S/Co. The sensitivity and specificity of Architect HBsAg assay are 99.80% and >99.5%, respectively (https://www.ilexmedical.com, accessed on 1 July 2022).

The qHBsAg titers were obtained by using the Elecsys HBsAg II assay (Roche Diagnostics, Mannheim, Germany), which is a two-step sandwich chemiluminescent microparticle immunoassay. The value was presented in IU/mL. HBsAg levels of >0.05 IU/mL are considered positive for HBV infection, and the lower limit of detection is 0.01 IU/mL. The qHBsAg result is calculated by automatically diluting sample 400 times and then calibrating the dilution factor. The positive range is 0.0 to 52,000 IU/mL and the sensitivity and specificity of the Elecsys HBsAg II assay are 100%, ranging from 99.7% and 99.8%, respectively [14,15,16].

HBeAg and anti-HBe and anti-HBV core (anti-HBc) antibody levels were measured by commercial immunoassays (Abbott Diagnostics) with the Architect i2000SR analyzer (Abbott Laboratories, Chicago, IL, USA) [17].

### 2.3. Statistical Analysis

Comparisons of continuous variables between the two groups were performed using the Student’s *t*-test, and categorical variables were compared using the chi-square test or Fisher’s exact test. The Spearman test was used to analyze the correlation between qHBsAg and sqHBsAg. The statistical analyses were performed using the R statistical programming environment (version 4.2.1; R development Core Team [http://www.R-project.org, accessed on 1 July 2022]), and *p*-values of <0.05 indicated statistical significance.

## 3. Results

### 3.1. All Samples

**Figure 1:**
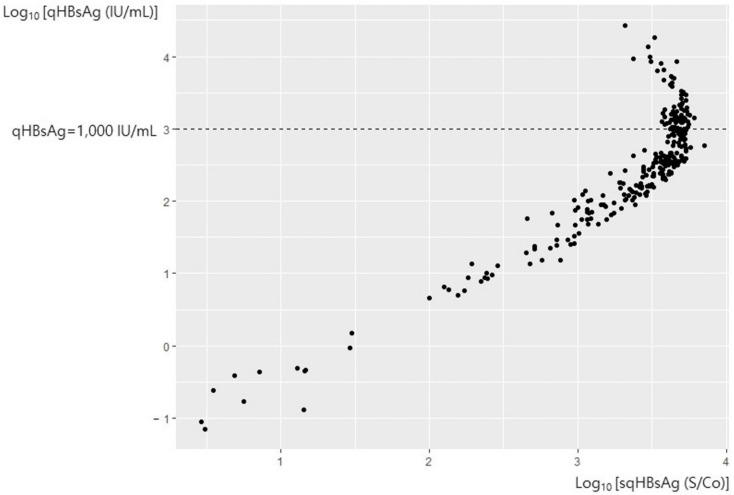
The correlation of sqHBsAg and qHBsAg in all samples. sqHBsAg, semi-quantitative hepatitis virus surface antigen; qHBsAg, quantitative hepatitis B virus surface antigen.

A total of 284 serum samples from patients with CHB were tested and quantitated for sqHBsAg and qHBsAg. When the patients were grouped according to the HBeAg status, 239 were HBeAg-negative and 45 were HBeAg-positive. Upon plotting the sqHBsAg and qHBsAg values, there was a significant hook effect when the qHBsAg value exceeded 1000 IU/mL (Figure 1). Thus, we separately analyzed samples according to the HBeAg status and evaluated correlations between sqHBsAg and qHBsAg.

### 3.2. Correlation of sqHBsAg and qHBsAg in HBeAg-Negative Chronic Hepatitis B

**Figure 2:**
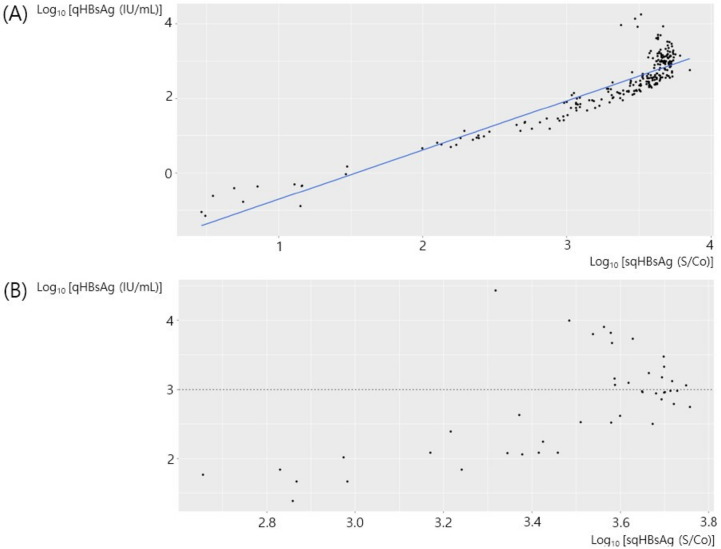
The correlation of sqHBsAg and qHBsAg in HBeAg-negative patients (**A**) and qHBsAg in HBeAg-positive patients (**B**). sqHBsAg, semi-quantitative hepatitis virus surface antigen; qHBsAg, quantitative hepatitis B virus surface antigen; HBeAg, hepatitis B envelope antigen.

The correlation between sqHBsAg expressed as S/Co and qHBsAg expressed as IU/mL in 239 samples with HBeAg-negativity is shown in Figure 2A. There was a linear correlation between sqHBsAg and qHBsAg and there was a minimal hook effect. There was a significant correlation between the two values (adjusted R^2^ = 0.8445, *p* < 0.001) and the qHBsAg [IU/mL] concentration could be expressed as 0.0094 × sqHBsAg [S/Co]^1.323^. We performed a subgroup analysis among the HBeAg-negative patients with qHBsAg > 1000 IU/mL. Although the statistical power was low, there was an inverse linear correlation between sqHBsAg and qHBsAg in this subgroup (adjusted R^2^ = 0.2950, *p* < 0.001).

### 3.3. Correlation of sqHBsAg and qHBsAg in HBeAg-Positive Chronic Hepatitis B

**Figure 3:**
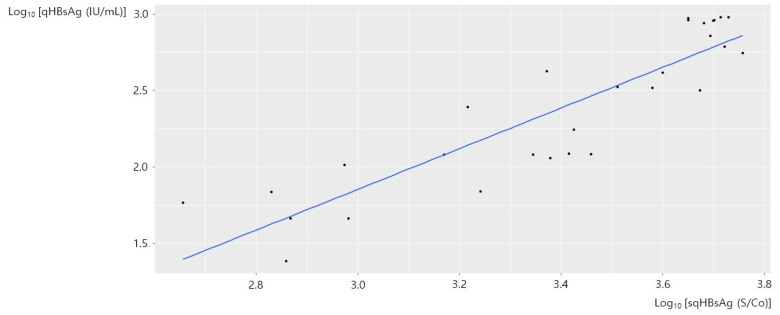
The correlation between sqHBsAg and qHBsAg in HBeAg-positive patients with qHBsAg < 1000 IU/mL. sqHBsAg, semi-quantitative hepatitis virus surface antigen; qHBsAg, quantitative hepatitis B virus surface antigen; HBeAg, hepatitis B envelope antigen.

**Figure 4:**
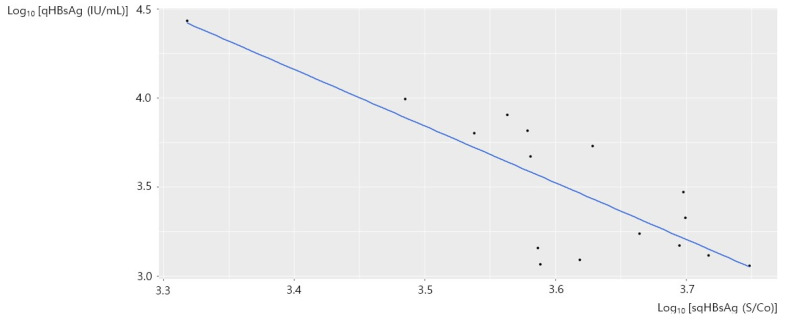
The correlatibetween sqHBsAg and qHBsAg in HBeAg-positive patients with qHBsAg ≥ 1000 IU/mL. sqHBsAg, semi-quantitative hepatitis virus surface antigen; qHBsAg, quantitative hepatitis B virus surface antigen; HBeAg, hepatitis B envelope antigen.

An inverse linear correlation between sqHBsAg and qHBsAg in 48 samples with HBeAg-positivity is shown in Figure 2B. We presumed that the inverse linear correlation was due to the hook effect, and we performed a stratified analysis with a qHBsAg cutoff value of 1000 IU/mL. Figure 3 shows the correlation between sqHBsAg and qHBsAg in 29 samples from HBeAg-positive patients with qHBsAg < 1000 IU/mL. There was a significant correlation between the two values (adjusted R^2^ = 0.7878, *p* < 0.001) and the qHBsAg [IU/mL] concentration could be expressed as 0.072 × sqHBsAg [S/Co]^1.331^. Figure 4 shows the correlation between sqHBsAg and qHBsAg in 19 HBeAg-positive patients with qHBsAg ≥ 1000 IU/mL. There was a significant inverse linear correlation between sqHBsAg and qHBsAg in this subgroup (adjusted R^2^ = 0.6350, *p* < 0.001) and qHBsAg [IU/mL] could be expressed as 8.987 × 10^14^ × sqHBsAg [S/Co]^−3.175^. Table 1 summarizes the correlation between sqHBsAg and qHBsAg.

## 4. Discussion

In this study, we investigated the correlation between sqHBsAg expressed as S/Co and qHBsAg expressed as IU/mL and found that there was a highly linear correlation between qHBsAg and sqHBsAg in HBeAg-negative CHB patients. There was a negative correlation between qHBsAg and sqHBsAg in HBeAg-positive CHB patients with a qHBsAg titer of ≥1000 IU/mL, which is consistent with the hook effect. 

HBV is a DNA virus that produces a series of viral protein products. Serologic and nucleic acid testing are critical for disease detection, prevention, and treatment. The information obtained from these tests not only helps to determine the infectious and immune status of the patient, guide appropriate monitoring strategies, and evaluate treatment efficacy; it also contributes to a better understanding of the epidemiology and natural history of the disease [18].

HBsAg is the first immunological marker to appear following acute HBV infection, and its early detection is critical to prevent disease transmission. Since the discovery of HBsAg in 1965, qualitative assays for HBsAg (considered as sqHBsAg in this study) have served as the standard test for HBV diagnosis in clinical practice [19,20]. Recently, there has been an increasing use of the qHBsAg assay in CHB as a predictor of response to treatment [21,22] and as a prognostic biomarker, [23] and qHBsAg is likely to be of increasing importance as it is applied in the individualization of HBV therapy. It is also probable that qHBsAg monitoring will be needed in future prospective trials of HBV treatments, and there is likely to be a growing need for reliable and easily accessible assays for qHBsAg. Finally, it has been suggested that serum qHBsAg should be used together with, but not as a substitute for, HBV DNA [12].

Previous studies have shown that qHBsAg measured by the Roche Elecsys HBsAg II assay correlates well with titers obtained by using the established Abbott Architect assay [24]. The measurement of qHBsAg has the potential to improve the reproducibility of the analysis while providing complementary information toward the deduction of the natural course of CHB; however, it is a relatively costly method that is not available in all areas. Therefore, in this study, we examined the correlation between sqHBsAg and qHBsAg and found that the results measured by a sqHBsAg method could be easily converted to qHBsAg values. Although the qHBsAg assay is widely used currently, this transformation can be used to leverage the results of sqHBsAg testing now as well as in the past.

Serum HBsAg levels have shown a strong correlation with other virological markers including serum HBV DNA and intra-hepatic cccDNA in the HBeAg-positive phase while showing a very weak correlation in the HBeAg-negative phase [25]. This dissociation between HBsAg production and HBV replication in HBeAg-negative patients may be partially explained by the different sources of HBsAg between the HBeAg-positive and HBeAg-negative phases. It appears that the HBsAg is mainly synthesized from cccDNA in HBeAg-positive patients, while HBsAg in HBeAg-negative patients has been traced to fragments of the HBV genome that have been integrated into the host chromosomes [26]. Accordingly, when we grouped our patients by HBeAg status, we found a highly linear positive correlation between qHBsAg and sqHBsAg in HBeAg-negative patients and a negative correlation between qHBsAg and sqHBsAg in HBeAg-positive patients.

One-step sqHBsAg immunoassays provide faster results by completing the reaction in a homogeneous system without washing steps. However, the hook effect occurs when excess antigen binds to the capture and detection antibodies, preventing an immune response and leading to underestimated or false-negative results [27]. Yang et al. have reported a hook effect-free qHBsAg chemiluminescence immunoassay, [27] and when we performed a stratified analysis by qHBsAg titer (≥ 1000 IU/mL) in HBeAg-positive CHB patients due to the hook effect, there was a negative correlation between qHBsAg and sqHBsAg.

There are several limitations in this study. First, the study cohort included a relatively small number of patients, especially in the HBeAg-positive group. Since we used real data from clinical practice, the majority of included patients were HBeAg-negative patients who may have a higher chance of HBsAg-seroclearance. In addition, although the statistical power was low due to the small sample size, the hook effect might be significant in HBeAg-negative patients with qHBsAg > 1000 IU/mL if the sample size was increased. Second, as the majority of Korean CHB patients are infected with genotype C2 HBV, [28,29] it is unclear whether our conversion formula can be applied to patients infected with HBV of other genotypes. Thus, multinational validation might be warranted.

In conclusion, there was a highly linear correlation between qHBsAg and sqHBsAg in HBeAg-negative CHB patients. Because of the hook effect, HBeAg-positive CHB patients with a high qHBsAg titer (≥1000 IU/mL) showed a negative correlation between qHBsAg and sqHBsAg. By using this correlation, sqHBsAg titers can be easily and reliably transformed to qHBsAg titers. Further large-scale studies may be needed to make maximal use of qHBsAg and to fully elucidate its role in clinical practice.

## Figures and Tables

**Table 1:** The correlation of sqHBsAg and qHBsAg.

**HBeAg (+)**								
sqHBsAg (S/Co)	100	500	1000	2000	3000	4000	1291.89	7286.87
qHBsAg (IU/mL)	3.32	28.27	71.11	178.91	306.91	450.09	100	1000
**HBeAg (−)**								
sqHBsAg (S/Co)	100	500	1000	2000	3000	4000	1108.73	6319.54
qHBsAg (IU/mL)	4.15	34.86	87.23	218.25	373.19	546.03	100	1000

sqHBsAg, semi-quantitative hepatitis virus surface antigen; qHBsAg, quantitative hepatitis B virus surface antigen; HBeAg, hepatitis B envelope antigen.

## Data Availability

The datasets used and/or analyzed during the current study are available from the corresponding author on reasonable request.

## References

[1] European Association For The Study Of The Liver (2012). EASL clinical practice guidelines: Management of chronic hepatitis B virus infection. J. Hepatol..

[2] Liaw Y.F., Kao J.H., Piratvisuth T., Chan H.L., Chien R.N., Liu C.J., Gane E., Locarnini S., Lim S.-G., Han K.-H. (2012). Asian-Pacific consensus statement on the management of chronic hepatitis B: A 2012 update. Hepatol. Int..

[3] Lok A.S., McMahon B.J. (2009). Chronic hepatitis B: Update 2009. Hepatology.

[4] Chan H.L., Wong V.W., Tse A.M., Tse C., Chim A.M., Chan H., Wong G.L., Sung J.J. (2007). Serum hepatitis B surface antigen quantitation can reflect hepatitis B virus in the liver and predict treatment response. Clin. Gastroenterol. Hepatol..

[5] Tseng T.-C., Liu C.-J., Yang H.-C., Su T.-H., Wang C.-C., Chen C.-L., Hsu C.-A., Kuo S.F.-T., Liu C.-H., Chen P.-J. (2013). Serum hepatitis B surface antigen levels help predict disease progression in patients with low hepatitis B virus loads. Hepatology.

[6] Tseng T.C., Liu C.J., Yang H.C., Su T.H., Wang C.C., Chen C.L., Kuo S.F., Liu C.-H., Chen P.-G., Chen D.-S. (2012). High levels of hepatitis B surface antigen increase risk of hepatocellular carcinoma in patients with low HBV load. Gastroenterology.

[7] Martinot-Peignoux M., Lapalus M., Asselah T., Marcellin P. (2014). HBsAg quantification: Useful for monitoring natural history and treatment outcome. Liver Int..

[8] Jaroszewicz J., Calle Serrano B., Wursthorn K., Deterding K., Schlue J., Raupach R., Flisiak R., Bock C.-Y., Manns M.P., Wedemeyer H. (2010). Hepatitis B surface antigen (HBsAg) levels in the natural history of hepatitis B virus (HBV)-infection: A European perspective. J. Hepatol..

[9] Lee J.H., Kim S.J., Ahn S.H., Lee J., Park Y., Kim H.S. (2010). Correlation between quantitative serum HBsAg and HBV DNA test in Korean patients who showed high level of HBsAg. J. Clin. Pathol..

[10] Kwon H.W., Lee H.Y., Kim S.G., Kim W., Jung Y.J., Kang K.W., Chung J.K., Lee M.C., Lee D.S. (2011). Quantitative measurement of serum hepatitis B surface antigen using an immunoradiometric assay in chronic hepatitis B. Nucl. Med. Mol. Imaging.

[11] Deguchi M., Yamashita N., Kagita M., Asari S., Iwatani Y., Tsuchida T., Iinuma K., Mushahwar I.K. (2004). Quantitation of hepatitis B surface antigen by an automated chemiluminescent microparticle immunoassay. J. Virol. Methods.

[12] Chan H.L.-Y., Thompson A., Martinot-Peignoux M., Piratvisuth T., Cornberg M., Brunetto M.R., Tillmann H.L., Kao J.-H., Jia J.-D., Wedemeyer H. (2011). Hepatitis B surface antigen quantification: Why and how to use it in 2011—A core group report. J. Hepatol..

[13] Liu Y.P., Yao C.Y. (2015). Rapid and quantitative detection of hepatitis B virus. World J. Gastroenterol..

[14] Liao C.C., Hsu C.W., Gu P.W., Yeh C.T., Lin S.M., Chiu C.T. (2015). Comparison of the elecsys HBsAg II assay and the architect assay for quantification of hepatitis B surface antigen in chronic hepatitis B patients. Biomed. J..

[15] Wursthorn K., Jaroszewicz J., Zacher B.J., Darnedde M., Raupach R., Mederacke I., Cornberg M., Manns M.P., Wedemeyer H. (2011). Correlation between the Elecsys HBsAg II assay and the Architect assay for the quantification of hepatitis B surface antigen (HBsAg) in the serum. J. Clin. Virol..

[16] Mühlbacher A., Weber B., Bürgisser P., Eiras A., Cabrera J., Louisirirotchanakul S., Tiller F.-W., Kim H.-S., Helden J.V., Bossi V. (2008). Multicenter study of a new fully automated HBsAg screening assay with enhanced sensitivity for the detection of HBV mutants. Med. Microbiol. Immunol..

[17] Kim M.A., Kim S.U., Sinn D.H., Jang J.W., Lim Y.S., Ahn S.H., Shim J.J., Seo Y.S., Baek Y.H., Kim S.G. (2020). Discontinuation of nucleos(t)ide analogues is not associated with a higher risk of HBsAg seroreversion after antiviral-induced HBsAg seroclearance: A nationwide multicentre study. Gut.

[18] Gish R.G., Locarnini S.A. (2006). Chronic hepatitis B: Current testing strategies. Clin. Gastroenterol. Hepatol..

[19] Lok A.S., McMahon B.J. (2007). Chronic hepatitis B. Hepatology.

[20] Blumberg B.S., Sutnick A.I., London W.T., Millman I. (1970). Australia antigen and hepatitis. N. Engl. J. Med..

[21] Brunetto M.R., Moriconi F., Bonino F., Lau G.K., Farci P., Yurdaydin C., Piratvisuth T., Luo K., Wang Y., Hadziyannis S. (2009). Hepatitis B virus surface antigen levels: A guide to sustained response to peginterferon alfa-2a in HBeAg-negative chronic hepatitis B. Hepatology.

[22] Moucari R., Korevaar A., Lada O., Martinot-Peignoux M., Boyer N., Mackiewicz V., Dauvergne A., Cardoso A.C., Asselah T., Nicolas-Chanoine M.H. (2009). High rates of HBsAg seroconversion in HBeAg-positive chronic hepatitis B patients responding to interferon: A long-term follow-up study. J. Hepatol..

[23] Tseng T.C., Kao J.H. (2013). Clinical utility of quantitative HBsAg in natural history and nucleos(t)ide analogue treatment of chronic hepatitis B: New trick of old dog. J. Gastroenterol..

[24] Louisirirotchanakul S., Khupulsup K., Akraekthalin S., Chan K.P., Saw S., Aw T.C., Cho D.H., Shin M.-G., Lim J. (2010). Comparison of the technical and clinical performance of the Elecsys HBsAg II assay with the Architect, AxSym, and Advia Centaur HBsAg screening assays. J. Med. Virol..

[25] Thompson A.J., Nguyen T., Iser D., Ayres A., Jackson K., Littlejohn M., Slavin J., Bowden S., Gane E.J., Abbott W. (2010). Serum hepatitis B surface antigen and hepatitis B e antigen titers: Disease phase influences correlation with viral load and intrahepatic hepatitis B virus markers. Hepatology.

[26] Mak L.Y., Seto W.K., Fung J., Yuen M.F. (2020). Use of HBsAg quantification in the natural history and treatment of chronic hepatitis B. Hepatol. Int..

[27] Yang R., Cui L., Liu Y., Cong X., Fei R., Wu S., Wei L. (2020). A hook-effect-free homogeneous light-initiated chemiluminescence assay: Is it reliable for screening and the quantification of the hepatitis B surface antigen?. Ann. Transl. Med..

[28] Song B.C., Cui X.J., Kim H. (2005). Hepatitis B virus genotypes in Korea: An endemic area of hepatitis B virus infection. Intervirology.

[29] Lee J.M., Ahn S.H., Chang H.Y., Shin J.E., Kim D.Y., Sim M.K., Hong S.P., Chung H.J., Kim S.O., Han K.H. (2004). Reappraisal of HBV genotypes and clinical significance in Koreans using MALDI-TOF mass spectrometry. Korean J. Hepatol..

